# Exploring the potential of *Bacillus* for crop productivity and sustainable solution for combating rice false smut disease

**DOI:** 10.3389/fmicb.2024.1405090

**Published:** 2024-05-28

**Authors:** Neha Pandey, Richa Vaishnav, Asha Singh Rajavat, Arvind Nath Singh, Sanjay Kumar, Ravi Mani Tripathi, Madan Kumar, Neeraj Shrivastava

**Affiliations:** ^1^Amity Institute of Microbial Technology, Amity University Uttar Pradesh, Noida, Uttar Pradesh, India; ^2^ICAR- Indian Institute of Seed Science, Maunath Bhanjan, Uttar Pradesh, India; ^3^ICAR- Indian Institute of Vegetable Research, Varanasi, Uttar Pradesh, India; ^4^Amity Institute of Nanotechnology, Amity University Uttar Pradesh, Noida, Uttar Pradesh, India; ^5^ICAR- Indian Institute of Agricultural Biotechnology, Garhkhatanga, Ranchi, Jharkhand, India

**Keywords:** *Bacillus*, bio-control, rice false smut, bio-formulation, *Bacillus vallismortis*, *Ustilaginoidea virens*, PGPR

## Abstract

Rice false smut, which is caused by the soil-borne fungal pathogen *Ustilaginoidea virens* (*U. virens*), is one of the most threatening diseases in most of the rice-growing countries including India that causes 0.5–75% yield loss, low seed germination, and a reduction in seed quality. The assessment of yield loss helps to understand the relevance of disease severity and facilitates the implementation of appropriate management strategies. This study aimed to mitigate biotic stress in rice by employing a rhizobacterial-based bioformulation, which possesses diverse capabilities as both a plant growth promoter and a biocontrol agent against *U. virens*. Rhizobacteria were isolated from the soil of the rice rhizospheres from the healthy plant of the false smut affected zone. Furthermore, they were identified as *Bacillus* strains: *B. subtilis* (BR_4), *B. licheniformis* (BU_7), *B. licheniformis* (BU_8), and *B. vallismortis* (KU_7) via sequencing. Isolates were screened for their biocontrol potential against *U. virens* under *in vitro* conditions. The antagonistic study revealed that *B. vallismortis* (KU_7) inhibited *U. virens* the most (44.6%), followed by *B. subtilis* BR_4 (41.4%), *B. licheniformis* BU_7 (39.8%), and *B. licheniformis* BU_8 (43.5%). Various biochemical and plant growth promoting attributes, such as phosphate and Zn solubilization, IAA, ammonium, siderophore, and chitinase production, were also investigated for all the selected isolates. Furthermore, the potential of the isolates was tested in both *in vitro* and field conditions by employing talc-based bioformulation through bio-priming and root treatment. The application of bioformulation revealed a 20% decrease in disease incidence in plants treated with *B. vallismortis* (KU_7), a 60.5% increase in the biological yield, and a 45% increase in the grain yield. This eco-friendly approach not only controlled the disease but also improved the grain quality and reduced the chaffiness.

## 1 Introduction

Rice (*Oryza sativa* L.), which belongs to the family Poaceae, is one of the highly demanded cereal crops not only in India but also worldwide. India is the first rice-growing country in the world in terms of cultivation area, which is approximately 42.5 million hectares with a production of 152.6 MT (Surendran et al., 2021). Most of the world’s population is dependent on this cereal crop and consumes it as a staple food after wheat (FAOSTAT, 2021; Statista, 2023). India is the second largest producer of rice in the world. In India, Assam, West Bengal, Odisha, Chhattisgarh, Jharkhand, and Bihar including Eastern Uttar Pradesh possess approximately 55% of the rice cropping area and contribute to approximately 50% of the total rice production (Mohanty and Yamano, 2017; Devkota et al., 2020). Approximately 85% of the rice farming system is focused on the Indo-Gagetic plains, with India occupying approximately 76% of this zone, including the state of Uttar Pradesh (Kumar et al., 2021).

**Figure 1:**
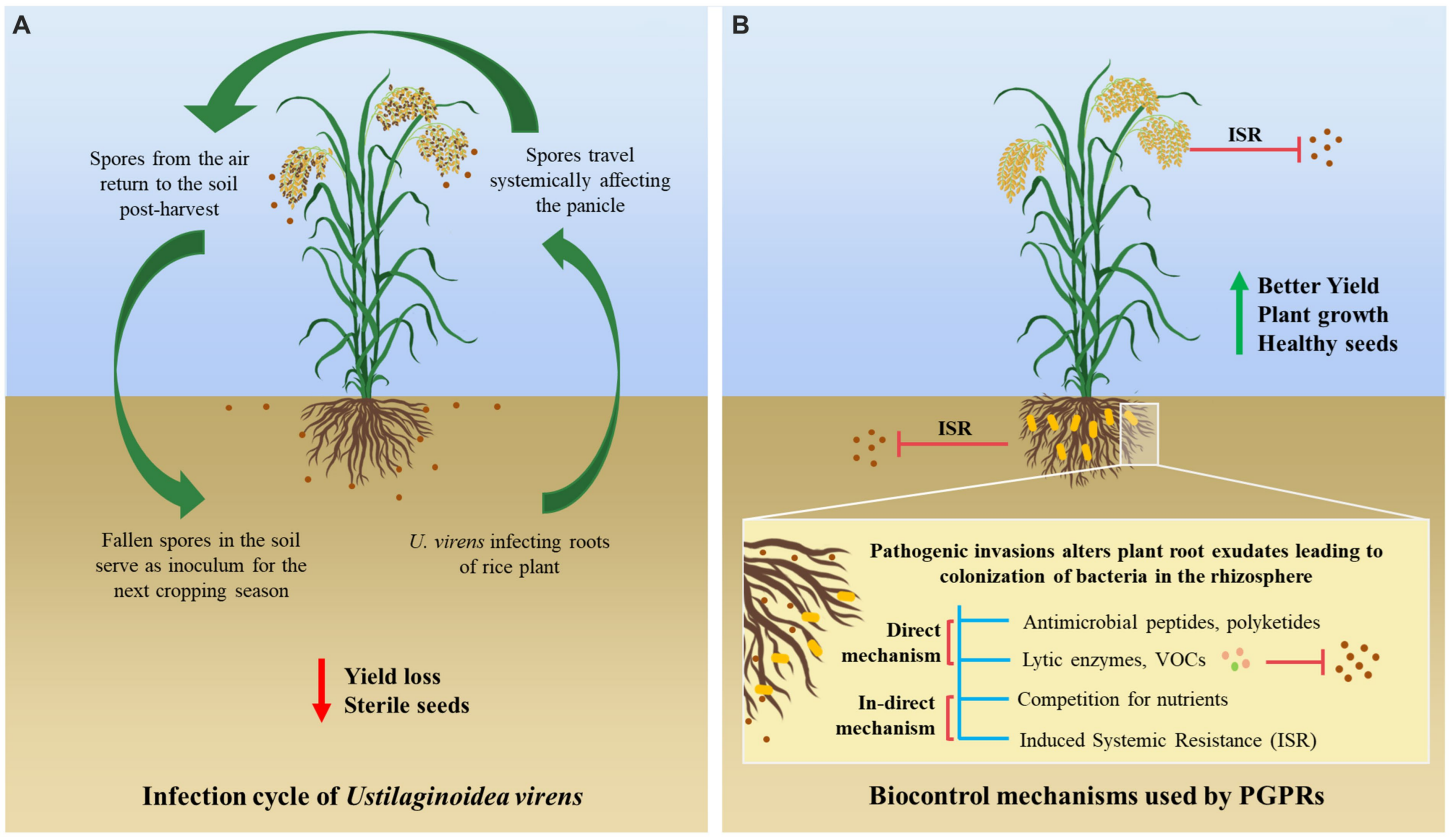
Schematic representation of **(A)** infection cycle of *U. virens* in rice plant leading to false smut and **(B)** mechanisms (direct and indirect) used by PGPRs in controlling disease.

The rice crop is challenged by various biotic and abiotic factors due to fluctuating environmental conditions in different geographical locations. A major biotic factor affecting rice cultivation is the rice false smut disease, which is one of the most devastating diseases caused by *Ustilaginoidea virens* or *Villosiclava virens* (teleomorph) (Chen et al., 2022). It severely affects the grain yield and the quality of rice, leading to 2.8–81% yield loss depending upon the severity of the infection. The chalkiness of the grains reduces the grain yield, and the infected seeds lose their seed viability (Muniraju, K. M. 2017; Yong et al., 2018). The pathogen travels systemically from the roots to the panicle of the rice plant, where it converts rice grains into yellow-green balls that contain mature chlamydospores, as it is primarily present in the soil. *U. virens* infects the stamen specifically, interrupting fertilization and flowering in rice and forming rice false smut balls (Fan et al., 2020). These rice false smut ball structures, which are known as pseudosclerotia, serve as an inoculum to spread disease in the neighboring rice plants under favorable climatic conditions, which infect the entire crop and the future crops (Tang et al., 2021) (Figure 1A).

To overcome the disease spread, the application of commercially available fungicides (trifloxystrobin with tebuconazole, propiconazole, and kresoxim-methyl) at distinct stages of rice growth, such as the booting stage, the panicle emergence stage, or the pre-flowering stage, is being utilized (Barnwal et al., 2016). However, incessant application of fungicides causes resistance in the pathogen against fungicides over a period of duration and persistence in the environment. Fungicides, such as propiconazole, have led to the development of fungicide-resistant *U. virens* strains due to repeated exposure, and also cause environmental pollution (Zhou et al., 2019). In addition to this, to enhance crop productivity and fulfil the global rice demand, farmers are employing the use of chemical fertilizers to enhance productivity as they are cost-effective and easily available. On the contrary, persistent use of chemical fertilizers decreases the nutritional value of seeds and its quality (Singh et al., 2014).

To alleviate the excessive use of fungicides and chemical fertilizers, there is a need to adapt to sustainable approaches in agricultural practices. To overcome these challenges, there is a need to use environmentally friendly and sustainable strategies that can serve as a biocontrol agent against the effect of biotic stress on the crop and a biofertilizer to enhance the yield (Sharma et al., 2022). In rice, microorganisms play a vital role in the exchange of plant nutrients and reduce the application of chemicals to a great extent (Jacoby et al., 2017). The use of microorganisms as bio-inoculants is the most alluring alternative to chemical-based fertilizers as they have a positive impact on plant growth, soil, and the environment (Kantachote et al., 2016; Jaiswal et al., 2022). The application of plant growth-promoting rhizobacteria (PGPR) can serve as a biocontrol, not only enhancing crop productivity but also aiding in the management of false smut disease.

Beneficial plant-microbe interactions in the rhizosphere can influence plant vigor and soil fertility (Gudeta, 2018). Rhizobacterial species, *Bacillus,* and fungal species, *Trichoderma,* have been widely used as potent biocontrol agents due to their secretion of toxic secondary metabolites that suppress or inhibit the growth of the pathogen (Baite et al., 2022). The application of PGPRs, such as *Pseudomonas* spp. (Guyasa et al., 2018), *Serratia* spp. (Nascente et al., 2019), *Actinomycetes* (Cruz et al., 2015), and various types of *Bacillus* species (Rais et al., 2018) on rice crops have been reported in various studies. These rhizospheric microbes can suppress plant pathogens through direct (antimicrobial peptides, production of chitinolytic enzymes, and volatile organic compounds, VOCs) and indirect (competition for nutrients and induction of resistance in the host) mechanisms (Shafi et al., 2017; Kohl et al., 2019) (Figure 1B).

In the present study, we have employed biocontrol PGPRs under greenhouse and field conditions to investigate the significant increase in disease management and plant agronomic parameters, as well as enhancement in grain quality. The genus *Bacillus,* the members of phylum *Bacillota,* are gram-positive, rod-shaped, aerobic bacteria. Their spore-forming ability enhances their application as bio-stimulants as they can survive in harsh and unfavorable environments and possess PGP traits (Vejan et al., 2016). The study investigates the role of multifarious biocontrol PGP bacteria, including *Bacillus licheniformis, Bacillus subtilis,* and *B. vallismortis,* isolated from the rice rhizosphere, in promoting growth, enhancing yield, and suppressing diseases in rice.

## 2 Materials and methods

### 2.1 Sampling and isolation of bacteria

Rhizospheric soil samples were collected from the five sites, namely, Takari, Dhata, Bhurchuni, Gopalpur, and Bamhrauli villages, of Fatehpur district, Uttar Pradesh, India (25.8500°N; 80.8987°E) (Supplementary Table 1). Samples were collected in triplicate from the healthy rice rhizosphere during the flowering stage when infection occurred and were pooled together. After the drying and sieving, soil samples were kept in a refrigerator at 4°C for further use. The rhizobacterial strains were isolated on nutrient agar (NA) media, soil extract agar, and kings B medium (HiMedia Ltd., India). By the serial dilution method, 1 g of soil was taken and dissolved in 10 mL of pre-sterilized distilled water. The dilution factors of 10ˉ^6^ to 10ˉ^8^ were taken for the isolation of bacteria and spread into nutrient agar, soil extract agar, and kings B medium amended Petri plates. Plates were incubated at 28 ± 2°C for 24 h. Single colonies were picked for obtaining pure culture (Gowsalya et al., 2014). A total of 135 colonies were picked from the Petri plate during the isolation of bacteria. For long-term preservation, bacteria were grown into Luria Broth (LB), and then 500 μL of broth was added into culture tubes. In total, 60% pre-sterilized glycerol was added into 2 mL screw top culture tubes. Tubes were previously stored at 4°C and then at 0°C followed by final storage at −80°C.

### 2.2 Screening of the collected bacterial isolates

#### 2.2.1 Based on biocontrol potential

For the preliminary screening of bacteria, all 120 rhizobacterial isolates were tested for the antagonistic activity against the phytopathogen. A dual culture test was performed against the rice false smut causative agent, *U. virens*. The fungal culture was obtained from the plant pathology laboratory of the ICAR-Indian Institute of Seed Science (IISS), Maunath Bhanjan, Uttar Pradesh, India. Strain *Ustilaginoidea virens* (NAIMCC-F-04112) was procured from the National Agriculturally Important Microbial Culture Collection (NAIMCC) of ICAR-National Bureau of Agriculturally Important Microorganisms, Maunath Bhanjan, Uttar Pradesh, India. To perform a dual culture test, a 5 mm disc of freshly grown fungal culture was placed on four sides of a Potato Dextrose Agar (PDA) Petri plate, and the bacterial isolate was inoculated at the center of the plate. Plates were incubated at the temperature of 28 ± 2°C for 10 days. Mycelial growth was measured following the method given by Kumar et al. (2012) and Maksimov et al. (2011).

The percentage inhibition was calculated using the following formula:


$$I=\frac{C-T}{C}\times 100$$

where *I* = percent of growth inhibition, C = diameter of fungal pathogen in control, and T = colony diameter/radial growth of the pathogen in treatment.

#### 2.2.2 Assessment of fungal inhibition by scanning electron microscope

A disintegration study of fungus mycelia was carried out using scanning electron microscopy (SEM) along with control. A portion of fungus near the inhibition zone was fixed in a 2.5% glutaraldehyde and incubated at room temperature (RT) for 60 min and stored in a freezer (4°C) for 12 h. Fungal samples were centrifuged at 5000 rpm for the removal of glutaraldehyde. Pallets were washed with 0.1 M phosphate buffer with pH 7 and dissolved in 0.1% silver nitrate solution for 1 h. Samples were dehydrated by using ethanol (30–90%) serially. The final sample was stored at 100% ethanol at 4°C for further study (Torres et al., 2016). This study was carried out using ZEISS EVO18 SEM with a magnification of X 4,000–5,000 X at the Instrumental facility of AIARS (Material and Devices), Amity University, Noida, India (Von Ardenne, 1985; Murtey and Ramasamy, 2021).

#### 2.2.3 Based on plant growth-promoting traits

PGP traits play a key role in regulating the growth of plants and defense system. PGPRs provide minerals and other key nutrients to plants.

##### 2.2.3.1 Phosphate solubilization

Phosphate solubilization was measured using a tricalcium phosphate (TCP) concentration of 3 gL^−1^ in an agar medium with a pH of 7.4 (Pikovskaya, 1948). A loopful of bacterial isolate was inoculated at the center of the Petri dish. All the plates were incubated at 28 ± 2°C for 7 days. A clear zone around a growing colony indicated phosphate solubilization. The halo zone was measured around the bacterial colony which determined phosphate solubilization activity.

##### 2.2.3.2 Indole acetic acid

To check the qualitative Indole Acetic Acid (IAA) production, isolates were cultivated in Luria Bertani broth supplemented with 100 mg/L of L-tryptophan as a precursor. Following 5 days of incubation, the cultures were centrifuged at 10,000 rpm for 10 min. The acquired supernatant was placed in a new tube, and Salkowski’s reagent was added in a 2:1 ratio. The solutions were gently mixed and kept at room temperature for 15–30 min to develop color. For the quantitative estimation (μg/ml), the standard plot of IAA was used (Farzana et al., 2009; Goswami et al., 2015).

##### 2.2.3.3 Siderophore solubilization

Qualitative production of siderophore was determined on Chrome Azurol S (CAS) agar medium. The isolates were spotted onto CAS agar plates and incubated at 28 ± 2°C for 6 days to observe the yellow to orange halo zone around the colony (Schwyn and Neilands, 1987).

##### 2.2.3.4 Zinc solubilization

To investigate the zinc solubilization efficiency of isolated cultures, bacterial isolates were spot inoculated in agar plates enriched by 1% Zinc oxide (ZnO) in the medium, which served as a source of insoluble Zn compound. Plates were incubated for 7 days at 28 ± 2°C in the dark condition to observe the transparent halo zone around the colony (Bunt and Rovira, 1955).

##### 2.2.3.5 Ammonium production

For the assessment of ammonium production, isolates were incubated in peptone water (10 mL) for 48–72 h at 28 ± 2°C. After incubation, Nessler’s reagent (0.5 mL) was added to each tube. The development of rich brown to faint yellow colors was observed as a result of ammonium activity (Cappuccino and Sherman, 1992).

##### 2.2.3.6 Chitinase production

To ascertain the amount of chitinase production test, 3 g of powdered chitin was dissolved in 75 mL of concentrated hydrochloric acid (HCl), and the mixture was then placed on a magnetic stirrer for an hour at room temperature. Subsequently, the solution was diluted with 1,000 mL of distilled water and filtered. To keep the colloidal chitin at pH 3.5, the residue was rinsed with distilled water twice. Spot inoculations of bacterial isolates were made in agar media enriched with chitin (0.3%), and the Petri plates were cultured at 28 ± 2°C for 6–7 days. Positive enzymatic activity was indicated by the formation of a clear zone (Krithika and Chellaram, 2016).

Furthermore, the remaining biochemical properties, including protease, cellulase, lipase, starch solubilization, urease test, and citrate utilization tests, were performed using the standard protocols as described by Cappuccino and Sherman (1992). An assessment of the ability to utilize different carbon sources and sugar utilization test was performed by the Hi25 Enterobacteriaceae Identification kit from HiMedia.

### 2.3 Molecular characterization and identification of the isolates

#### 2.3.1 16S rRNA sequencing

The molecular characterization of selected isolates was performed by 16S rDNA gene sequencing. DNA of freshly grown (48 h) bacterial isolates was extracted using a HiPurA® Multi-Sample DNA Purification Kit (MB554) of HiMedia. Polymerase Chain Reaction (PCR) mixture (25 μL) was prepared by adding 1 μL template DNA and universal primers 27F (5-AGAGTTTGATCMTGGCTCAG-3′) and 149R (5- CGGTTACCTTGTTACGACTT-3′) (Tan et al., 1997; Abellan-Schneyder et al., 2021). PCR cycle condition was performed as follows: 94°C for 3 min, 35 cycles of 94°C for 30 s, 50°C for 30 s,72°C for 90/60 s; final extension, 72°C for 7 min; and final hold at 4°C. The PCR product was resolved in ethidium bromide amended 1% agarose gel electrophoresis and visualized on a gel doc transilluminator (Quantity One, Bio-Rad, USA). Products were purified and used for genetic sequencing. The gene sequencing was outsourced to Eurofins Genomics India Pvt. Ltd., Bangalore.

#### 2.3.2 Identification and phylogenetic analysis

Sequence results were compared using the Basic Local Alignment Search Tool (BLASTn) program on the National Center for Biotechnology Information (http://www.ncbi.nlm.nih.gov), and accession numbers were obtained (Thompson et al., 1997). Phylogenetic analysis was conducted using MEGA XI software. The neighbor-joining (NJ) DNA distance technique was used to reconstruct evolutionary history. The evolutionary distances were expressed in base substitutions per site and were calculated using the maximum composite likelihood technique. The analysis involved 14 nucleotide sequences which were taken from the National Centre for Biotechnology Information (NCBI) database. A total of 1,000 bootstrap resampling tests were done to determine the branching percentage value (Felsenstein, 1985; Saitou and Nei, 1987; Kumar et al., 2018; Tamura et al., 2021).

### 2.4 Preparation of talc-based bioformulation

A loopful of isolated bacteria [*Bacillus subtilis* (BR_4); *B. licheniformis* (BU_7); *B. licheniformis* (BU_8); and *B. vallismortis* (KU_7)] were inoculated in 400 mL of pre-sterilized nutrient broth medium. The broth was incubated in a shaker incubator at 150 rpm for 72 h at 28 ± 2°C. The broth contained a bacterial load of 9 × 10^8^ cfu ml^−1^ after incubating for 3 days (Karthiba et al., 2010; Shah et al., 2021). A total of 1 kg of sterilized talc powder (121°C/15 lbs) was amended with 400 mL of bacteria-loaded nutrient broth for 1 h. Then, 1.5% calcium carbonate was added to maintain the pH to neutral, and 10% carboxymethyl cellulose (CMC) was added for the stickiness and used as an adhesive element. Bioformulations were air-dried in aseptic conditions and moisture was maintained at 20%. After the drying, bioformulations were stored in a refrigerator at 4°C in a sterilized polythene cover. Before the bio-priming of the seed, the viability of the formulation was checked. A total of 10 g of bioformulation was used for the 1 kg of seeds biopriming.

### 2.5 Effect of bacterial strains on seed germination

After verifying the antagonistic activity test, further evaluation of the plant growth promotion test was performed in *in vitro* conditions. The seed emergence test was carried out by the standard ‘Ragdoll paper’ method proposed by the International Seed Testing Association (ISTA 2007). Talc-based bioformulation with a bacterial load of up to 10^6^–10^8^ was used for the bio-priming of the rice seeds. A total of two hundred seeds with four replications were examined for seed emergence (germination percentage). Seedling root length, shoot length, seedling’s fresh weight, and dry weight were taken for the analysis of the vigor index and were taken on the 14^th^ day.

The seedling vigor index was calculated by using the formula (Abdul-Baki and Anderson, 1973):


$$\mathrm{Vigor}\;\mathrm{Index}\;I=\mathrm{Seedling}\;\mathrm{length}\;\left(\begin{array}{l} \mathrm{shoot}\;\mathrm{length}+\\ \mathrm{root}\;\mathrm{length} \end{array}\right)\times \;\mathrm{germination}\%$$

VigorIndexII=Seedlingdrywt.×germination%

After the measurement of seedling length, roots were removed very carefully from the shoot for the root architecture study. The root was floated on the surface of the root scanner machine Epson Expression 12000XL A3 Flatbed Photo Scanner, India. The study was conducted at the fungal study department of ICAR-National Bureau of Agriculturally Important Microorganisms, Maunath Bhanjan, Uttar Pradesh, India.

### 2.6 Efficacy of isolates under greenhouse conditions

Surface sterilized (5% sodium hypochlorite) seeds treated with *Bacillus* isolates [control, BR_4; BU_7; BU_8; and KU_7] were shown in pots. The plastic pots with a 10 kg capacity were filled with sterilized soil and sand in a ratio of 2:1. A total of 10 seeds of each treatment were directly sown in the pots. This experiment is performed in triplicate with three sets. After the seeding pots were filled with distilled water, extra plants were uprooted after the germination on 25–28^th^ day. Plant height, number of tillers, and other agronomical parameters were recorded following the standard protocol (Unkovigich et al., 2010). In total, 1,000 seeds weight were taken after the 150 days by the full harvest. Furthermore, field trials with the same isolates were conducted consecutively for 2 years for further analysis with the same isolates.

### 2.7 Field study/trials

**Figure 2:**
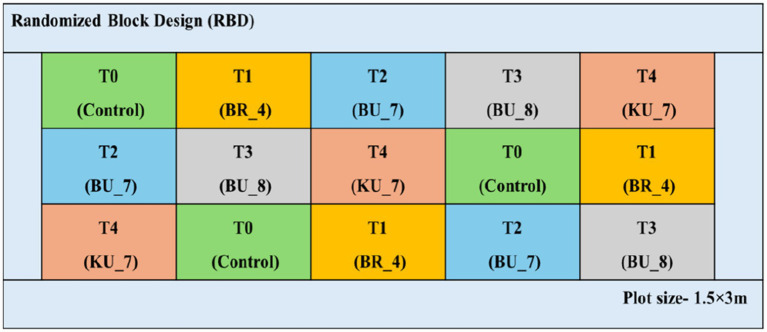
Experimental plot of various treatments under the field conditions (2019–2020) and field layout (RBD) of the treatments in ICAR-IISS, Maunath Bhanjan.

Previously, we have conducted seed biopriming techniques to evaluate the isolates in controlled conditions. In field conditions, direct seed sowing is not possible for paddy seeds, thus we have considered the root treatment techniques for further analysis. The evaluation of plant growth promoting (PGP) isolates was carried out by the root treatment technique with the HKR-126 rice cultivar. This cultivar is highly susceptible to the *U. virens* (Figure 2).

The field experiment was conducted consecutively for 2 years (2019 and 2020) in the field of ICAR-Indian Institute of Seed Science, Kushmaur, Maunath Bhanjan, Uttar Pradesh, India (25°56′30″N 83°33′40″E). The mean of maximum and minimum temperatures in both years were recorded between 36°C and 17°C, from June to October. Annual precipitations were recorded in the range of a maximum of 103.6 mm to a minimum of 14 mm. Four isolates were taken for the treatment, [C-control versus BR_4-root treatment with *Bacillus subtilis* (BR_4); BU_7-root treatment with *B. licheniformis* (BU_7); BU_8-root treatment with *B. licheniformis* (BU_8); and KU_7- root treatment with *B. vallismortis* (KU_7)]. For this experiment, 25 days old seedlings were taken for root treatment. Bioformulation was taken in the ratio of 10 g/L. Seedling roots were soaked overnight to facilitate colonization in the area surrounding the roots. Following that, three replications of seedlings were transplanted into the field. In this experiment, Randomized block design (RBD) was employed. Plot sites measured 1.5 × 3 m, with a plant-to-plant and row-to-row spacing of 15 and 20 cm, respectively. Various agronomical parameters were recorded till the harvesting periods, including numbers of tillers and plant height in 60 days after transplanting (DAT), panicle length, flag leaf length, and width on 100 DAT; and finally, biological yield and total grain yield and 1,000 seeds weight were measured after full harvest on 145^th^ DAT (Figure 2; Supplementary Figure S1).

### 2.8 Study of pathogenicity

#### 2.8.1 Fungal inoculation preparation

For the pathogenicity test, the fungal inoculum of the pathogen was prepared in the laboratory. A 10 mm disc of *U. virens* mycelia was inoculated into a 250 mL potato sucrose broth medium and kept in a shaker incubator at 150 rpm and 28 ± 2°C for 15 days. After the filtration, spores were harvested. Spore counting was performed using the hemocytometer under the compound microscope at a magnification of 40X. After the spore count, desirable dilutions (2 × 10^6^ spores/ml) were made for future tests. The filtered spore suspension was used as an inoculum (Bhunjun et al., 2021; Baite et al., 2022).

#### 2.8.2 Disease incidence

During the experiment, the disease incidence of all experimental plots (4.5 m^2^/replication) was recorded. The observation was taken after the full flowering and seed maturation as the disease incidence occurs after the flowering after 90 DAT.

The percentage of infection data was calculated using the standard formula (Baite et al., 2020a,b):


$$\mathrm{Disease}\;\mathrm{Incidence}\;\left(\%\right)=\frac{\mathrm{Number}\;\mathrm{of}\;\mathrm{infected}\;\mathrm{tillers}}{\mathrm{Total}\;\mathrm{number}\;\mathrm{of}\;\mathrm{tillers}}\times 100$$

### 2.9 Evaluation of photosynthetic pigment, protein, and antioxidants

#### 2.9.1 Estimation of total chlorophyll

For the estimation of total chlorophyll, 0.5 g of the fresh leaf sample was crushed and extracted into 10 mL of 80% chilled acetone. Samples were centrifuged at 5000 RPM for 10 min, and supernatants were taken for the analysis. The absorbances of extracts were observed at 645, 663, and 470 nm. Chlorophyll content was calculated using the following formula (Arnon, 1949).


$$\mathrm{Chlorophyll}\;a=\;\left[\left(12.7\;x\;A\;663\right)-\left(2.69\;x\;A\;645\right)\right]\text{;}$$

$$\mathrm{Chlorophyll}\;b=\;\left[\left(22.9\;x\;A\;645\right)-\left(4.68\;x\;A\;663\right)\right]\text{;}$$

$$\mathrm{Carotenoids}=\;\left[\begin{array}{l} \left(1000\;x\;A\;470\right)-\\ 3.27\;x\;\mathrm{Chlorophyll}\;a+\\ \mathrm{Chlorophyll}\;b \end{array}\right]/227$$

#### 2.9.2 Estimation of proteins

Total Protein Extraction Kit (BCBSP003) (BR Biochem Life Sciences, Pvt. Ltd., New Delhi, India) was used for total protein quantification. The experiment employed 0.5 g of fresh leaf sample, and the absorbance of the sample was measured at A260/A280 nm using Nanodrop (Thermo Scientific-2000C).

#### 2.9.3 Superoxide dismutase

A total of 1 g of leaf sample was frozen and dried in liquid nitrogen to prevent proteolytic activity. The sample was ground in 10 mL of extraction buffer containing 0.1 M phosphate buffer, pH 7.5, 0.5 mM EDTA, and 1 mM ascorbic acid. The extract was run through four layers of cheesecloth, and the filtrate was centrifuged for 20 min at 15,000 rpm. The supernatant was utilized for the analysis of the superoxide dismutase enzyme (Dhindsa et al., 1981).

#### 2.9.4 Catalase

Using the standard protocol of Aebi (1984), a catalase assay was performed. The reaction mixture (3 mL) contained 0.5 mL of 75 mM H_2_O_2_ and 1.5 mL of 0.1 M phosphate buffer (pH 7). The reaction was started by adding 50 μL of diluted enzyme extract. A UV–visible spectrophotometer (Specord Bio-200, Analytik Jena, Germany) was used for the analysis of enzymatic activity. The absorbance was taken at 240 nm using the UV spectrophotometer.

### 2.10 Statistical analysis

The experiments were conducted in triplicates, and the obtained results were expressed in terms of the mean ± standard deviation. Duncan’s multiple range tests (DMRT) were used for the data analysis (Duncan, 1955). Field data were analyzed using one-way and two-way ANOVA, where *, **, and *** represent significant and **** represents highly significant at *p* ≤ 0.05, 0.01, 0.001, and 0.0001, respectively. Statistical analysis was performed by using IBM SPSS Statistic 27 software and GraphPad Prism version 8.0.2(263) software.

## 3 Results

### 3.1 Site selection and isolation of PGPRs

Soil samples were collected during the kharif crop season from the rice fields of Fatehpur districts of Uttar Pradesh, India. During the collection of samples, special care was taken in selecting the rice plants and only those plants that were at the late booting stage, vigorous, and were not affected by any kind of disease were considered. From the collected rhizospheric soil samples, a total of 120 rhizobacterial isolates were selected based on their different colony morphologies, colors, and texture variations.

### 3.2 Screening of PGPRs

#### 3.2.1 Based on biocontrol potential against *Ustilaginoidea virens*

**Figure 3:**
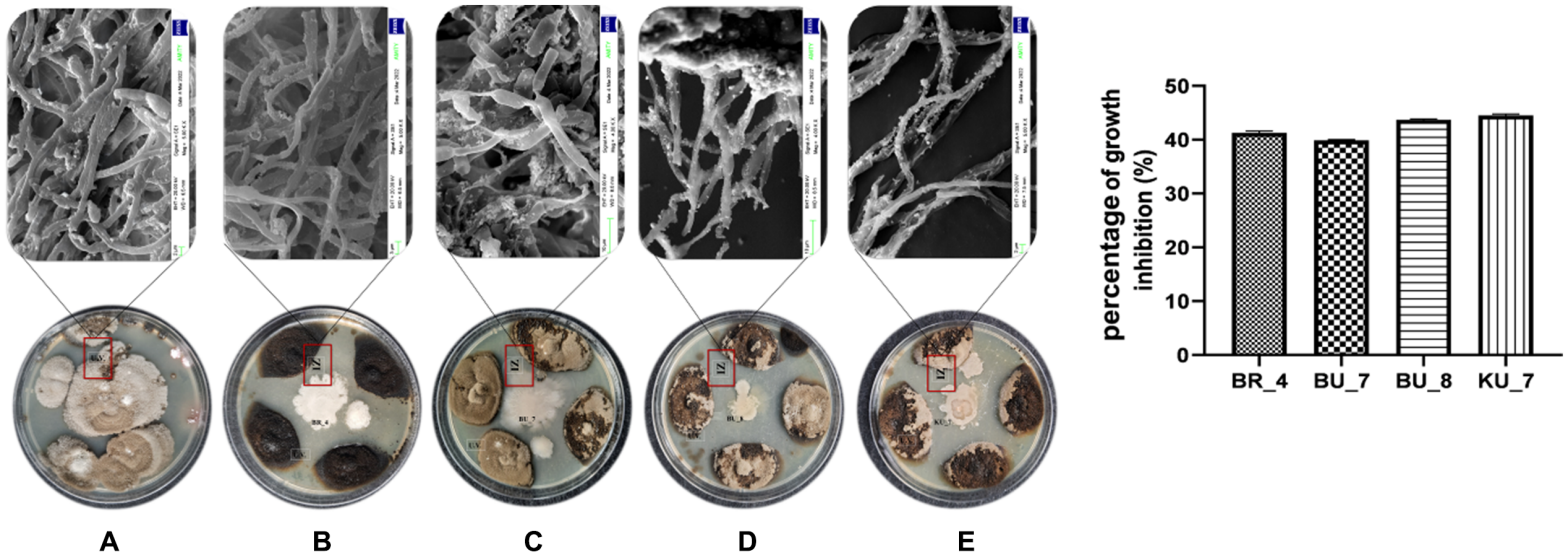
Rhizobacterial isolates showing antifungal activity against the *U. virens* containing the fungal Inhibition Zone (IZ) with mycelial degradation study using Scanning Electron Microscopy. **(A)** Control (*U. virens*). **(B)** BR_4 *Bacillus subtilis*. **(C)** BU_7 *B. licheniformis*. **(D)** *B. licheniformis* BU_8. **(E)** KU_7 *B. vallismortis*. **(F)** Percentage of growth inhibition by the rhizobacteria.

Furthermore, all the selected isolates were tested against *U. virens* to assess the biocontrol properties of the isolates. Based on the dual culture assay, maximum growth inhibition of the false smut pathogen was shown by four isolates (BR_4, BU_7, BU_8, and KU_7), and those isolates were chosen for further study. Isolate KU_7 showed the maximum inhibition zone at 44.6% and BU_7 recorded a minimum of 39.8% (Figure 3). The altered morphology of mycelia was observed around the inhibition zone during biocontrol assay. To eliminate this altered morphology or lethality of bacteria against the fungi, the mycelia were observed under SEM (Scanning Electron Microscope). The results showed that all the bacteria disintegrated the mycelia and disrupted the intact mycelia into pieces (Figure 3).

#### 3.2.2 Assessment of plant growth promoting traits

After selecting the potent microbes, we further assessed the plant growth-promoting traits, such as phosphate solubilization efficiency, Indole-3-Acetic Acid (IAA) production, siderophore production, Zn solubilization efficiency, ammonium production, and chitinase production, which are very crucial factors for plant growth and fitness along with soil health. Table 1 shows the results of PGP traits along with their effectiveness.

**Table 1:** Plant growth promoting traits of isolated rhizospheric bacterial strains.

Strains	Phosphate assay (mm)	IAA (μg/mL)	Zn Solubilization	Siderophore assay	Ammonium production	Chitinase assay
BR_4	5.3 ± 0.06	10.5 ± 0.01	−	+	+	+
BU_7	2.1 ± 0.04	6.4 ± 0.07	−	++	+	+
BU_8	−	7.5 ± 0.02	−	++	++	+
KU_7	1.0 ± 0.07	1.6 ± 0.03	++	−	+	+

IAA-Indole acetic acid; −, no activity; W, weak activity; +, activity; ++, very good activity.

Among all four isolates, BR_4 showed the maximum phosphate solubilization activity on Pikovskaya’s agar medium by forming a halo zone around their colonies. The diameter of the halo zone was recorded at 5.3 mm (the highest) in BR_4, and the least was recorded in BU_7 at 1.1 mm. In the case of Indole-3-Acetic Acid (IAA) production, BR_4 isolate produced 10.5 μg/mL, and the minimum was observed in KU_7, which is 1.6 μg/mL. To assess the siderophore production ability, the test was carried out for all the isolates, and three isolates were found to be positive for siderophore production. Among all four isolates, only KU_7 showed Zn solubilization efficiency, which is a very essential component for increasing native Zn availability in soil, which led to increased assimilation of Zn in the host. The selected isolates were also very good chitin solubilizers. In addition to that, we have also performed starch, ammonium utilization, cellulase, lipase, catalase, and urease activity tests. The results are shown in Table 2. After assessing the plant growth-promoting traits, the next phase was to identify the species of bacteria.

**Table 2:** Biochemical characterization of isolated rhizospheric bacterial strains.

Strains	Protease	Cellulase	Lipase	Starch	Urease	Citrate
BR_4	−	+	+	++	+	−
BU_7	−	+	+	++	++	+
BU_8	−	+	+	−	++	++
KU_7	−	+	+	+	+	−

−, no activity; W, weak activity; +, activity; ++, very good activity.

### 3.3 Identification of the selected biocontrol PGPRs

#### 3.3.1 16S rRNA sequencing

With the help of the 16S rRNA sequencing of the FASTA file database, bacterial isolates were identified. Bacterial sequencing data were submitted to the NCBI GenBank to get the accession numbers, BR_4 *B. subtilis* MZ356383, BU_7 *B. licheniformis* MZ350854, BU_8 *B. licheniformis* MZ356392, and BU_7 *B. vallismortis* MZ359961. Based on 16S rDNA sequence analysis, all four isolates were identified as *Bacillus* genera with different species. Percentage homology, sequence lengths, and accession numbers of all four isolates are summarized in Table 3.

**Table 3:** Molecular characterizations of bacterial isolates, BLAST results showing sequence length, percentage of homology, and accession number given by NCBI.

Isolate code	Sequence length (bp)	Query cover (%)	E Value	% of identity	Sequence Id	Accession no.	Post Identification
BR_4	694	100	0.0	99.89	SUB9806821	MZ356383	*Bacillus subtilis*
BU_7	898	100	0.0	99.71	SUB9806808	MZ350854	*B. licheniformis*
BU_8	695	100	0.0	99.13	SUB9806827	MZ356392	*B. licheniformis*
KU_7	651	100	0.0	97.06	SUB9813921	MZ359961	*B. vallismortis*

#### 3.3.2 Phylogenetic analysis

**Figure 4:**
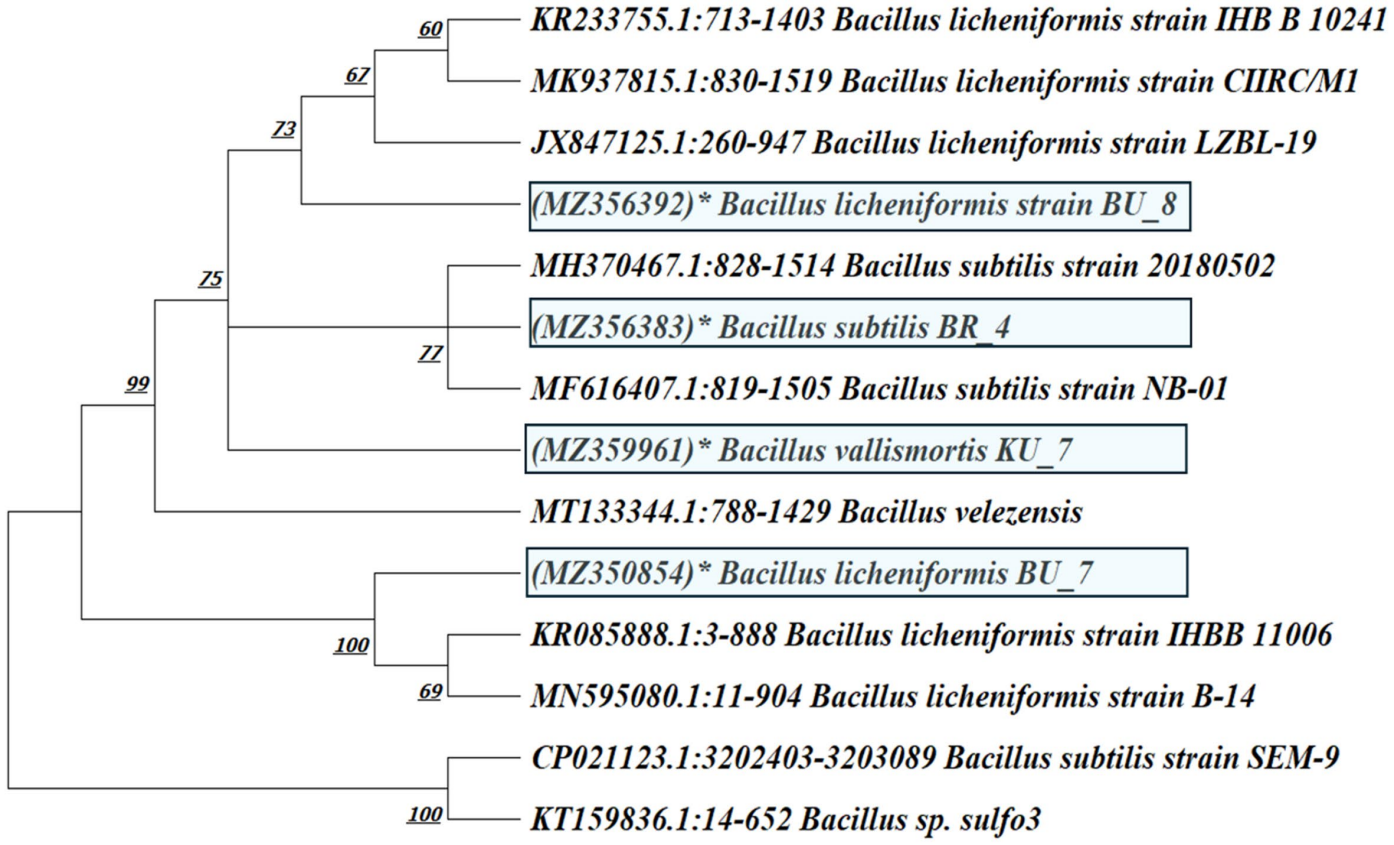
Phylogenetic tree of *B. subtilis* (BR_4), *B. licheniformis* (BU_7 and BU_8), and *B. vallismortis* (KU_7) based on evolutionary neighbor-joining (NJ) relationships.

Phylogenetic analysis was conducted with MEGA XI software. The analysis involved 14 nucleotide sequences. A total of 1,000 bootstrap resampling tests were done to determine the branching percentage value. The accession numbers of isolates are mentioned in brackets and are shown in Figure 4.

### 3.4 Effect of bacterial treatment on growth characteristics under *in vitro* condition

#### 3.4.1 Effect of bioformulation on germination

In this experiment, seeds were bio-primed with bioformulation prepared by using all four selected isolates. To prepare the bioformulation, an efficient carrier, and an inert base were used, i.e., talc powder containing magnesium silicate. It is widely accepted and utilized in the biofertilizer industry. Furthermore, the shelf life of talc-based bioformulation was checked up to 180 days. For the viability test and contamination check, bioformulation was tested routinely on every 30th day found. Till 180th days after inoculation (data unpublished).

To verify the effect on seed germination and related physiological parameters in the presence of bacterial isolates, we used bio-primed rice seed with bioformulations having bacterial loads of up to 10^6^–10^8^. After the 14^th^ day inoculation, we examined the results and observed that all the treatments BU_7, BU_8 (*B. licheniformis*), BR_4 (*B. subtilis*), and KU_7 (*B. vallismortis*) resulted in enhanced physiological parameters such as seed emergence percentage, root length and shoot length, seedling fresh weight, and seedling dry weight, vigor index I, and vigor index II. Seed germination increased by 16% in the treatment KU_7 (98.7), and 15% in the treatment BU_8 (97.67) as compared to the control (82.8). In treatment BU_7, it was recorded by 12% (94.67), and in BR_4, emergence was enhanced by 11% (93.17) (Table 4).

**Table 4:** Effect of bacterial inoculation on physiological parameters and vigor of plants.

Treatment	Germination (%)	Root length (cm)	Shoot length (cm)	Seedling fresh wt. (mg)	Seedling dry wt. (mg)	Vigor index I	Vigor index II
C	82.83 ± 0.29^d^	7.3 ± 0.35^d^	9.2 ± 0.06^d^	108.6 ± 0.58^d^	61.7 ± 0.35^d^	1369.5^d^	5.1^d^
BR_4	93.17 ± 0.29^c^	10.9 ± 0.55^c^	11.2 ± 0.35^c^	148.6 ± 0.58^c^	101.7 ± 0.59^c^	2059.0^c^	9.5^c^
BU_7	94.67 ± 0.29^b^	11.4 ± 0.15^b^	13.3 ± 0.38^b^	170.5 ± 0.59^b^	116.5 ± 0.60^b^	2347.7^b^	11.0^b^
BU_8	97.67 ± 0.58^a^	12.7 ± 0.12^a^	14.3 ± 0.21^a^	188.4 ± 0.55^a^	120.8 ± 0.31^a^	2643.5^a^	11.8^a^
KU_7	98.07 ± 0.58^a^	13.1 ± 0.12^a^	15.4 ± 0.21^a^	195.2 ± 0.55^a^	128.9 ± 0.31^a^	2784.4^a^	12.6^a^

a, b, c denotes significant treatments, where (a) is highly significant, (b) is medium significant, and (c) is least significant. Different letters are significantly different by Duncan’s multiple range test (DMRT). Values are expressed in means ± standard deviation (*n* = 5), the significance of ANOVA *p* < 0.05.

Seeds treated with KU_7 (*B. vallismortis*) showed the maximum root and shoot length, which were observed at 13.1 cm and 15.4 cm, respectively. The minimum root and shoot length were observed with treatment BR_4 (BR_4) *B. subtilis,* which were observed at 10.9 cm and 11.2 cm. The uninoculated control root length was recorded as 7.3 cm and the shoot length was 9.2 cm, which is smaller than all the bio-primed treatments.

After the 14^th^ day of treatment, the seeds that emerged in ragdoll paper were safely extracted, and fresh and dry weights were observed in all the treatments. Maximum fresh weight and dry weight were recorded in treatment KU_7, showing 195.2 mg and 128.9 mg weight, respectively. Control seedling fresh and dry weights were recorded as the least, with 108.6 mg for fresh and 61.7 mg for dry. All the isolates were higher in all the parameters compared to the control, and among them, KU_7 was found superior to other isolates. Vigor index I (VI-I) and vigor index II (VI-II) were observed in all the treatments. Treatment KU_7 showed high VI-I (2784.4) and VI-II (12.6), while the treatment BR_4 (*B. subtilis*) showed a minimum vigor index, VI-I (2059) and VI-II (9.5). The VI-I and VI-II of control seedlings were recorded the least, with 1369.5 and 5.1, respectively.

#### 3.4.2 Effect of bioformulation under greenhouse conditions

After obtaining promising results in germination experiments, furthermore, to observe the promotional growth effect of bacterial isolates, the pot experiments were performed under controlled environmental conditions in three replications. During the experiment, several attributes, such as root parameters, plant height, the number of tillers, flag leaf length and width, panicle length, and 1,000 seed weight, were recorded.

**Figure 5:**
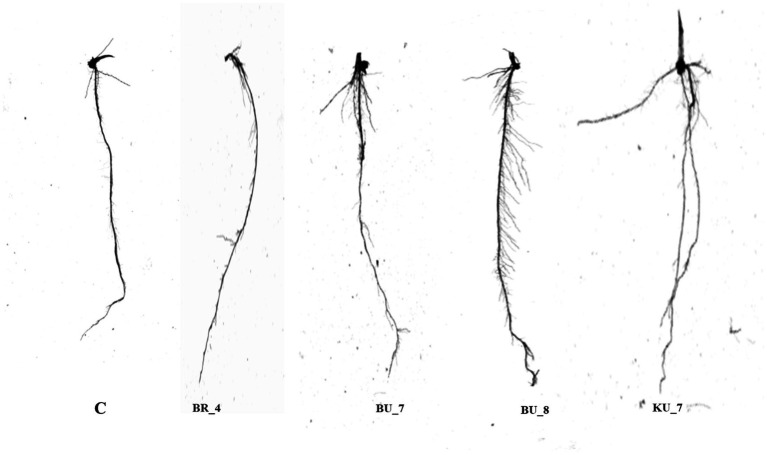
Variations study in root system architecture after inoculation of the different bioinoculants (C) Control, (BR_4) *Bacillus subtilis*, (BU_7) *B. licheniformis*, (BU_8) *B. licheniformis*, *and* (KU_7) *B. vallismortis.*

First, a root architecture study was performed to analyze the effect of bioagents on the root growth and physiological parameters of the root system. For this purpose, roots from 28-day-old rice seedlings were carefully removed from the pot, thoroughly washed with distilled water, and transferred to the root scanner for examination. All the treatments were found to be superior in root parameters as compared to the control root. The results showed that the inoculation of KU_7 (*B. vallismortis*) changed the root architecture of the seedlings and exhibited a significant increase in total root length, number of root tips, total surface area, and root volume as compared to other treatments and control (Figure 5). Table 5 shows the comparable representation of all observed root parameters.

**Table 5:** Effect of bacterial inoculation on root architecture study.

Treatment	Total length	Total Surface Area (cm^2^)	Average Diameter (mm)	No. of Root Tips	Root Volume (cm^3^)	Root length (cm)		
						Diameter 0.0–0.50 (mm)	Diameter 0.50–1.00 (mm)	Diameter >1.00 (mm)
C	36.85 ± 7.16^e^	4.23 ± 0.60^d^	0.37 ± 0.02^d^	96.00 ± 8.72^e^	0.02 ± 0.01^e^	7.88 ± 0.96^d^	0.70 ± 0.33^d^	0.23 ± 0.11^e^
BR_4	78.87 ± 10.17^c^	8.50 ± 1.09^a^	0.42 ± 0.06^d^	119.33 ± 10.97^d^	0.02 ± 0.01^d^	9.98 ± 0.55^cd^	1.54 ± 0.58^c^	0.37 ± 0.29^d^
BU_7	57.19 ± 9.95^d^	7.57 ± 0.76^b^	0.49 ± 0.04^d^	124.33 ± 12.42^c^	0.02 ± 0.01^d^	10.81 ± 0.87^c^	2.34 ± 1.12^a^	0.50 ± 0.36^b^
BU_8	96.23 ± 13.15^b^	7.66 ± 1.45^b^	0.53 ± 0.10^b^	141.00 ± 32.05^b^	0.04 ± 0.22^d^	18.24 ± 1.81^a^	2.29 ± 0.60^ab^	0.43 ± 0.36^c^
KU_7	127.74 ± 10.30^a^	7.11 ± 0.26^c^	0.51 ± 0.2^c^	160.33 ± 1.53^a^	0.14 ± 0.01^c^	15.71 ± 0.51^b^	1.94 ± 1.33^c^	0.42 ± 0.14^d^

Values are expressed in means ± standard deviation (*n* = 5), significance of ANOVA *p* < 0.05. Data represented with different alphabets are significantly different from each other, where (a, b) are highly significant, (c) is medium significant, and (d, e) are least or non-significant.

Furthermore, all other agronomical parameters, including number of tillers, plant height, flag leaf length, panicle length, and 1,000 seed weight, were recorded at different phases of the pot trial study. Full harvesting was carried out on the 145^th^ day at the maturation stage.

On the 65th day following the transplant, plant height and number of tillers were observed. In the experiment, the highest plant height was observed at 111.8 cm in treatment KU_7, while the lowest was 105.8 cm in treatment BR_4. The height of the control plant was recorded 95.3 cm. During the tiller count, the treatment KU_7 recorded with the maximum number of tillers (11.0 tiller/plant), whereas the treatment BR_4 with the minimum tillers were observed (9.00 tiller/plant), while the control was recorded 6 tiller/plant.

Plant height, flag leaf length and width were noted after the 60^th^ day, during the heading stage. Treatment BU_8 recorded the highest flag leaf length (FLL) and width (FLW), which was recorded at 33.8 cm for FLL and 1.9 cm for FLW. Control depicted the lowest 24.5 cm for FLL and 0.90 cm for FLW. Panicle length was taken as thin during the 100th day. A significantly high panicle length was observed in the treatments (KU_7) and (BU_8), which was recorded at 20.1 cm and 19.2 cm, respectively, while the C-control represented the lowest panicle length of 10.3 cm.

Table 6 The application of PGPR corresponding to higher 1,000 seeds weight in treatment (KU_7) was recorded as 24.9 g/1000 seeds, and the lowest was recorded in treatment with (BR_4) with 22.9 g/1000 seeds. Control was recorded at 18.8 g/1000 seeds.

**Table 6:** Effect of bacterial inoculation on agronomical parameters and 1,000 seeds weight.

Treatment	Plant height (cm)	No. of tillers	Flag leaf length (cm)	Flag leaf width (cm)	Panicle length (cm)	1,000 seeds (g)
C	95.3 ± 0.76^c^	6 ± 0.58^c^	24.5 ± 0.47^c^	0.90 ± 0.06^c^	10.3 ± 0.42^c^	18.8 ± 0.76^d^
BR_4	105.8 ± 0.83^b^	9 ± 0.58^c^	30.0 ± 0.25^c^	1.6 ± 0.06^c^	16.3 ± 0.42^b^	22.9 ± 0.81^c^
BU_7	106.1 ± 0.84^a^	11 ± 0.58^a^	31.9 ± 0.38^b^	1.8 ± 0.0^b^	18.5 ± 0.31^a^	23.6 ± 0.49^a^
BU_8	109.8 ± 0.80^a^	10 ± 0.50^b^	33.8 ± 0.10^a^	1.8 ± 0.06^a^	19.2 ± 0.20^a^	24.1 ± 0.57^b^
KU_7	111.8 ± 0.80^a^	11 ± 0.50^b^	34.6 ± 0.10^a^	1.9 ± 0.06^a^	20.1 ± 0.20^a^	24.9 ± 0.57^b^

Values are expressed int means ± standard deviation (*n* = 5), with significance of ANOVA *p* < 0.05. a, b are significant treatments, where (a) is highly significant, (b) is medium significant, and (c, d) are non-significant or carrying the least value.

### 3.5 Biochemical analysis

#### 3.5.1 Photosynthetic pigments and protein content analysis

**Figure 6:**
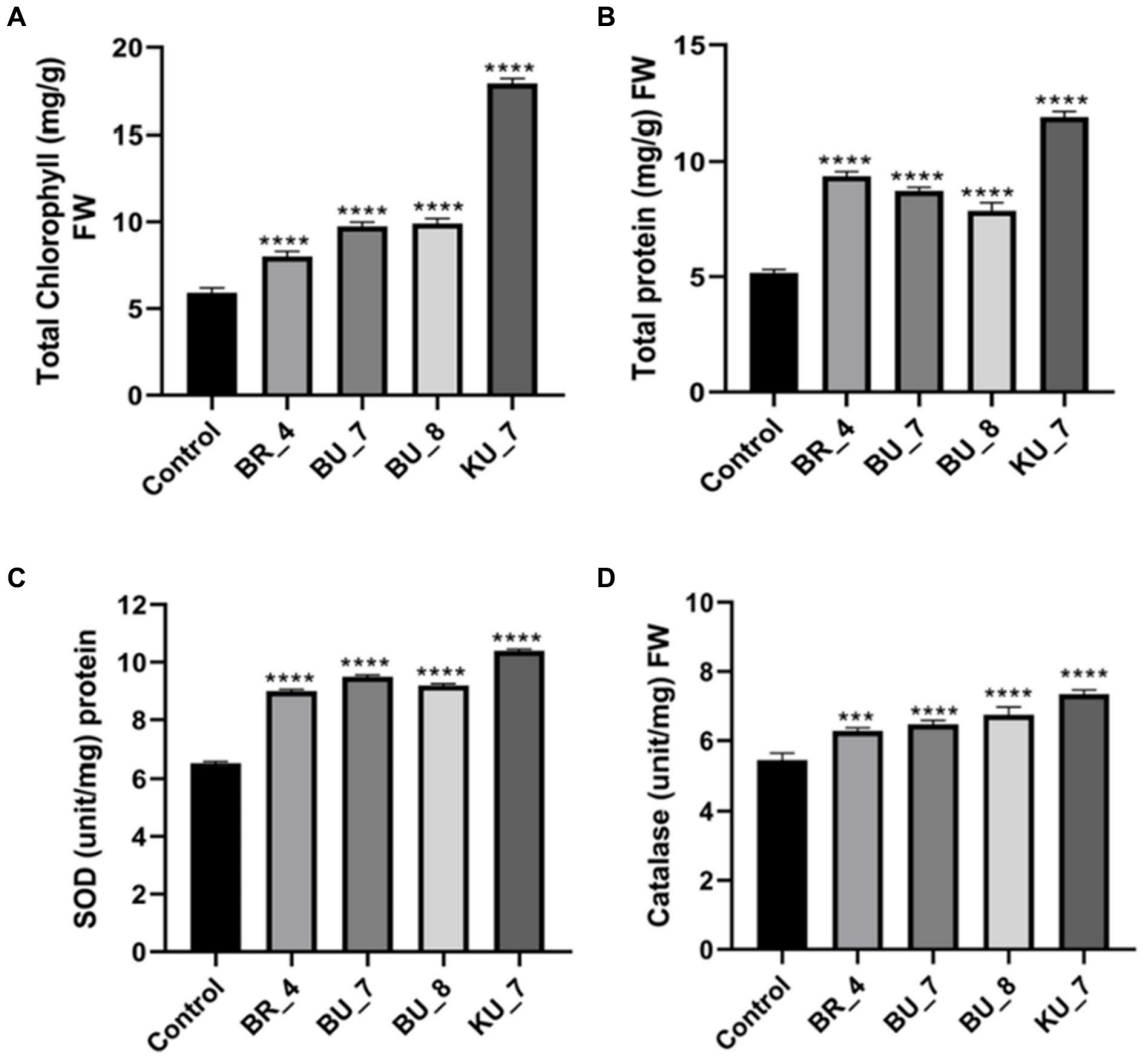
Effect of treatments on photosynthetic pigment, protein content, and antioxidant enzymes. **(A)** Total chlorophyll (mg/g) FW. **(B)** Total protein(mg/g) FW. **(C)** Superoxide dismutase (SOD) (Unit/mg) protein. **(D)** Catalase (unit/mg) FW. The tests were analyzed using one-way ANOVA where *, **, and *** represent significance and **** high significance at *p* ≤ 0.05, 0.01, and 0.001 and 0.0001, respectively.

The total chlorophyll content of treated seedlings of all the treatments were recorded. The total chlorophyll content was recorded at a maximum of 17.9 mg g^−1^ fresh weight (FW) of leaves in KU_7 (*B. vallismortis*), and then treatment (BU_8) *B. licheniformis* (9.9 mg g^−1^) FW were significantly affected (*p* < 0.05) by the bio-inoculation in comparison to control (5.9 mg g^−1^) FW (Figure 6A). The maximum value of total protein content was observed to be 11.87 mg g^−1^ FW in treatment (KU_7) *B. vallismortis,* while minimum was observed in treatment (BR_4) *B. subtilis* (9.37 mg g^−1^) FW. The rice plant without any treatment (control) has a protein content of 5.17 mg g^−1^ (Figure 6B).

#### 3.5.2 Evaluation of antioxidant enzyme activity

To assess the stress-responsive parameters or defense-related parameters, catalase and superoxide dismutase (SOD) activities were analyzed during the late booting stage. SOD is an important constituent of the antioxidant mechanism against oxidative stress, and the maximum was observed to be 10.40 U mg^−1^ (protein) in treatment KU_7, and a minimum was observed to be 9.0 U mg^−1^ (protein) in BR_4. Control plants recorded the least SOD activity, i.e., 6.5 U mg^−1^ (protein) (Figure 6C). Furthermore, the CAT activity shows a similar pattern, as already shown by the SOD enzyme. The CAT activity was assayed in the experiment, and the maximum was observed in KU_7, i.e., 7.37 U mg^−1^ FW, and the minimum was observed in BR_ 6.28 U mg^−1^ FW. The untreated control was measured at only 5.44 U mg^−1^ FW, which was the least as compared to the treated plants (Figure 6D).

### 3.6 Effect of bioinoculants under field conditions

Furthermore, to verify the efficacy of bacterial isolates in the enhancement of rice growth and yield, the field trial was conducted in the Eastern region of Uttar Pradesh during the kharif season (from June to October). Rice seedlings were treated with bacterial isolates in the experimental field by using the standard protocol. Rice was grown for 145 days till the maturity phase, then harvested and examined for various growth and yield parameters. This study is based on the results of the 2 years of field trials conducted in the years 2019 and 2020 (Figure 2; Supplementary Figure S1).

**Figure 7:**
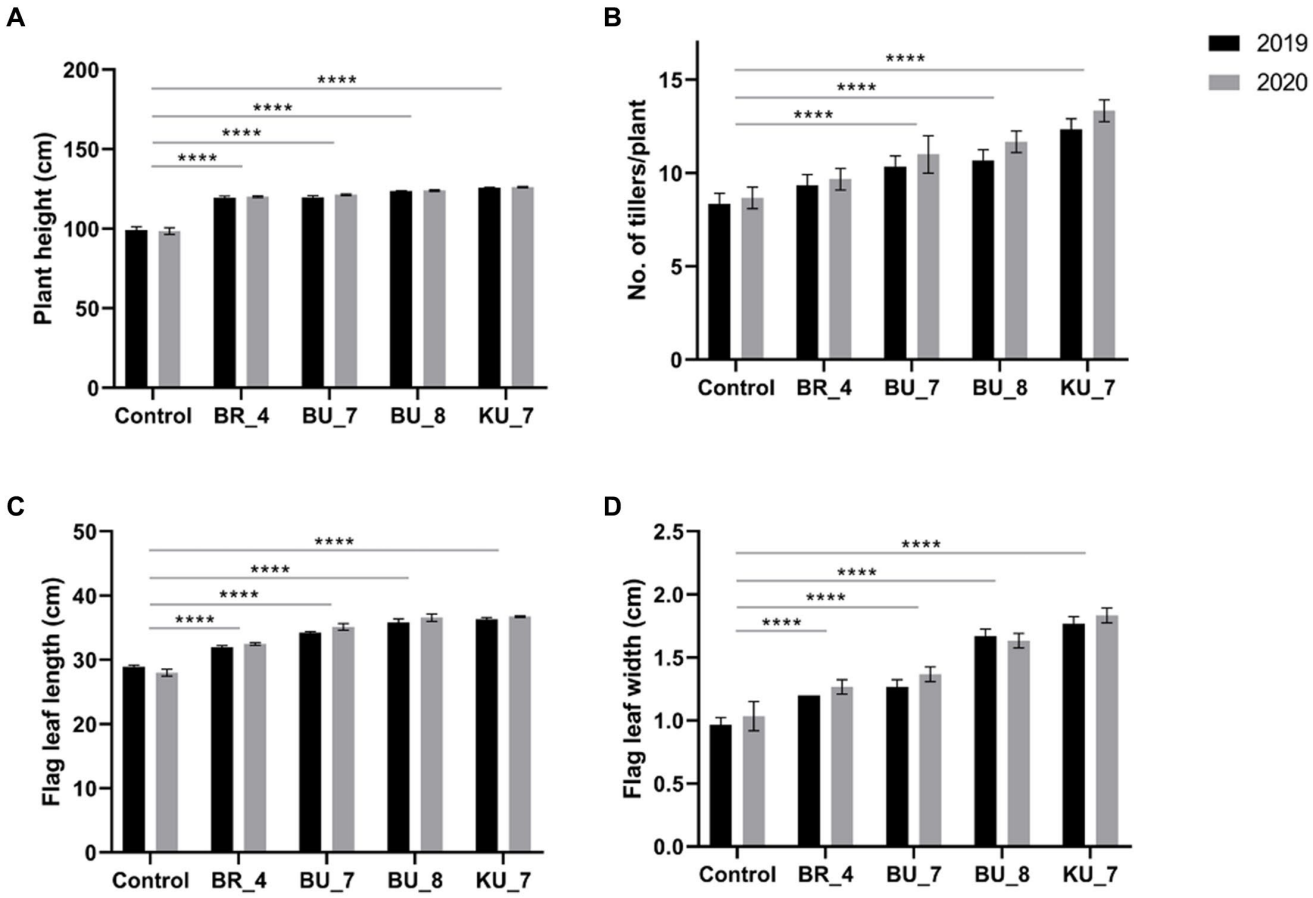
Effect of root treatment on agronomical parameter of rice under field conditions. **(A)** Plant height (CM). **(B)** Number of tillers /plants. **(C)** Flag leaf length (cm). **(D)** Flag leaf width (cm). The tests were analyzed using Two-way ANOVA where *, **, and *** represent significance and **** high significance at p ≤ 0.05, 0.01, and 0.001 and 0.0001, respectively.

After 60 days of sowing, at the heading stage, the plant height was measured. The data is represented in Figure 7A, and it was observed that treatment KU_7 showed the highest height in both trial years 2019 and 2020 with 125.8 cm and 126.2 cm, respectively. When this was compared with control field plants, it was observed that the height of the KU_7 field plant was 26% higher in 2019 and 28% higher in 2020. Moreover, the lowest height was found in treatment BR_4 with a length of 119.4 cm in the year 2019 and 120.1 cm in 2020, which was increased by 20% in both years, while control was measured at 99.2 cm in the first year (2019) and 98.5 cm in the second year (2020) during the experiment (Figure 7A).

The number of tillers was counted during the late booting stage of cropping. The data for 2019 and 2020 for tiller numbers were observed to be maximum in treatment KU_7 with 13.3 tillers/hill in 2019 and 12.3 tillers/hill in 2020, which was increased by 48 and 52%, respectively. The lowest number of tillers was recorded in treatment BR_4 with 9.3 tillers/hill in the first year (2019) and 9.7 tillers/hill in the second year (2020), which were increased by 12 and 11.5%, respectively. The tiller count in control was recorded at 8.3 tillers/hill in the first year and 8.7 tillers/hill in the second year (Figure 7B).

Flag leaf plays a major role in crop production as it aids in the majority of photosynthetic activity, which provides a larger amount of photosynthate to plant for grain filling. The seedlings treated with the (KU_7) observed a significantly high flag leaf length (FLL) and flag leaf width (FLW). FLL was recorded as 36.1 cm in the first year and 36.7 cm in the second year, which was non-significant with each other in both years but significant when compared with other treatments and control. The lowest FLL was recorded in (BR_4) with 32.0 cm for the first year and 32.5 cm for the second year, while the control was depicted as 28.6 cm in 2019 and 28. 7 cm in 2020 (Figure 7C). FLW was recorded as higher in treatment (KU_7) with 1.7 cm in 2019 and 1.8 cm in 2020. Here, the same pattern was observed, similar to FLL. The graph shows the lower width in treatment (BR_4) with 1.2 cm in the first year, and 1.3 cm in the second year, while the control was recorded at 1.0 cm in both years. (Figure 7D).

**Figure 8:**
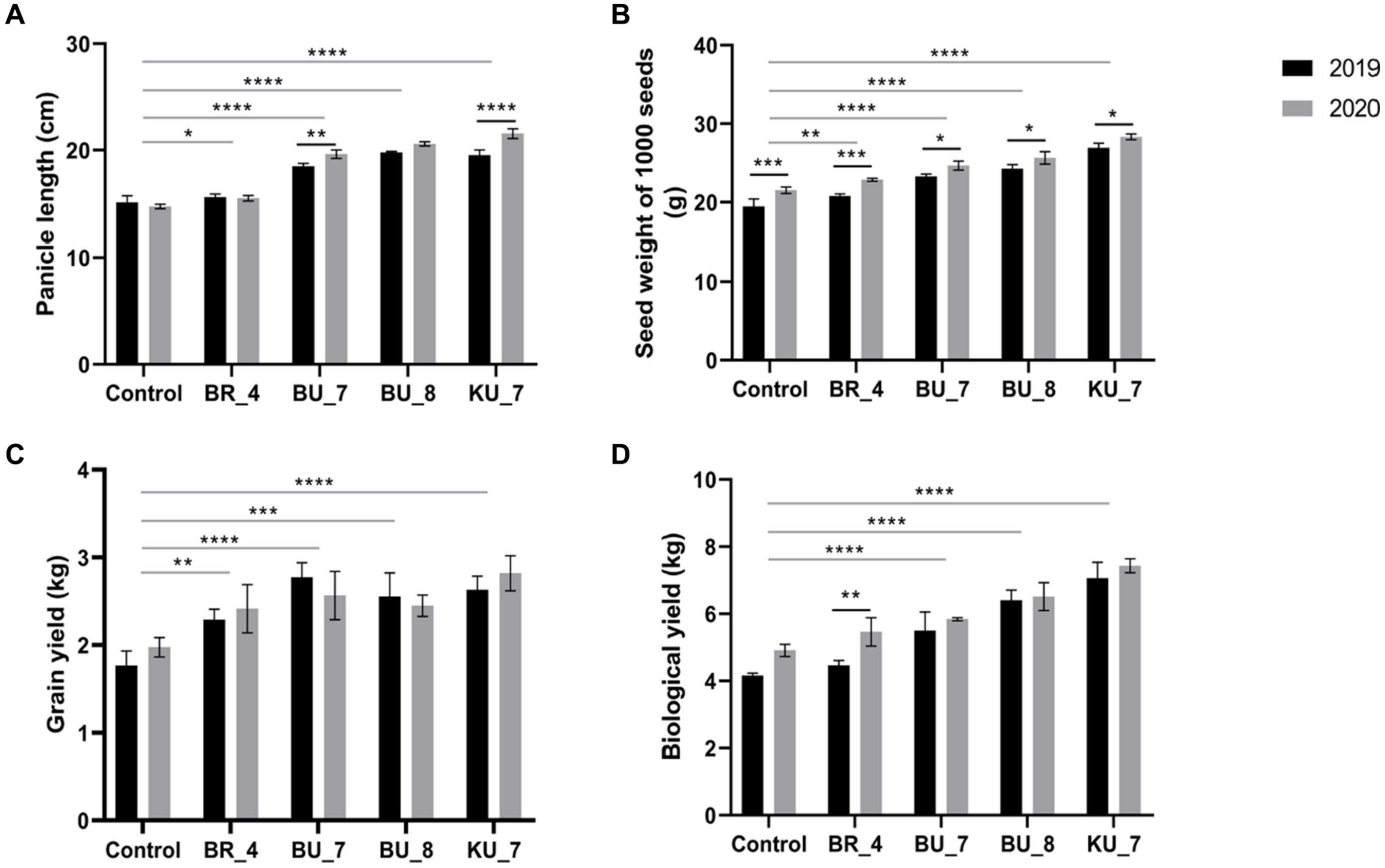
Effect of root treatment on agronomical parameter of rice under field conditions. **(A)** Panicle length. **(B)** 1,000 seeds weight. **(C)** Grain yield. **(D)** Biological yield. The tests were analyzed using Two- way ANOVA where *, **, and *** represent significance and **** high significance at p ≤ 0.05, 0.01, and 0.001 and 0.0001, respectively.

The data for 2019 and 2020 showed significantly high panicle length in both years. Figure 8A indicates the panicle length data, which is the highest for KU_7 with a length of 19.5 cm in 2019 and 21.6 cm in 2020 and the growth increased by 29 and 46% in 2019 and 2020, respectively. The panicle length of all the treatments was higher as compared to the untreated control plant, which had the lowest panicle length in both years, i.e., 15.1 cm and 14.7 cm, respectively (Figure 8A).

The application of PGPR by the root application resulted in a corresponding significant increase in 1000 seed weight in treatment KU_7 with 26.93 g in 2019 and 28.33 g per 1,000 seeds in 2020, which were increased by 31.6 and 38.3%, respectively. The lower weight appeared in treatment (BR_4) with 20.80 g in 2019 and 22.87 g/1000 seeds in 2020, which were increased by 6.8 and 6.2%, respectively, while the least data were recorded in control with 19.47 g in the first year of the experiment and 21.52 g/1000 seeds in the second year. All treatments with the PGPRs showed significantly higher seed weight compared to the control in both years of the experiment (Figure 8B).

Total yield and biological yield were calculated after the harvesting of crops. The two years of field trial data indicated that *B. vallismortis* (KU_7) gave a significantly higher biological yield in both the years 2019 and 2020. Biological yield in the first year was recorded at 7.06 kg and in the second year, it significantly increased and was recorded at 7.43 kg in KU_7. It increased by 69.71% in (2019) and 51.32% in (2020), which is the highest among all the treatments. In the case of grain yield, it was recorded at 2.63 kg in the first-year experiment and 2.82 kg in the second year in KU_7. Total grain yield increased by 48.8% in 2019 and 42.4% in 2020 (Figure 8C).

The biological yield was observed to be minimum in treatment with *B. subtilis* (BR_4), in which the biological yield was recorded at 4.46 kg in 2019, which was increased by 7%, and 5.46 kg in 2020, which was increased by 9.2%. The grain yield was measured to be 2.29 kg in 2019 and 2.42 kg in 2020. Total grain yield increased by 29 and 22.2%, respectively.

The biological yield and grain yield were recorded as minimum in control plants, i.e., 4.16 kg in 2019 and 4.91 kg in 2020 and 1.77 kg in 2019 and 1.98 kg in 2020, respectively (Figure 8D).

### 3.7 Disease incidence

The field experiment was transplanted into a false smut-infested field, which was highly affected by the rice false smut disease and caused more than 50% yield loss in previous years. A total of 10 plants/replications (total of 30 plants/treatment) were marked for the artificial inoculation of fungal pathogens during the late booting stage. Our isolates showed antagonistic activity against the *U. virens,* which resulted in lower disease incidence found in (KU_7) *B. vallismortis* and *B. subtilis* (BR_4) and was observed between 5 and 10% DSI range. Disease incidence in treatment (BU_8) *B. licheniformis* was recorded at 18%, and higher smut balls were observed in treatment (BU_7) *B. licheniformis* and showed 20% of disease incidence. Control was observed with the highest number of infected panicles and depicted 40% of disease incidence. Control seeds lost their quality and weight. More chaffiness occurred after the final harvest.

## 4 Discussion

Rice (*Oryza sativa* L.) is one of the most important crop which is cultivated worldwide. It plays a significant role in global food consumption by people around the world (Xu et al., 2021). Due to changes in climatic conditions and geographical location, the crop is challenged by various biotic and abiotic stresses (Vaishnav et al., 2014; Hussain and Mahmood, 2020). Among which, one of the main concern is the rice false smut disease caused by *U. virens,* posing a major threat to rice production. Rice false smut leads to major economic losses and poses a threat to global food security as it not only reduces the yield of the crop but also leads to seed sterility, reduction in grain size, and milling quality, affecting productivity in the subsequent season. It affects rice panicles, forming masses of greenish-black spores and produces mycotoxins, which pose health risks to humans (Laha et al., 2016; Bag et al., 2021). Taking into consideration the aforementioned perspective, this study utilized biocontrol properties of the Plant Growth-Promoting Rhizobacteria (PGPR) to control false smut infection in rice plants through root treatment and their potential role in disease management strategy. The use of microbes is a sustainable way to overcome biotic stress along with maintaining soil health and fitness.

Here, we have employed the rhizospheric bacteria that belong to *Bacillus* species, i.e., *B. licheniformis*, *B. vallismortis,* and *B. subtilis*. These bacteria are isolated from the various regions of Fatehpur, Uttar Pradesh, India, which were severely affected and marked regions of *U. virens* infections (Pandey et al., 2024) (Supplementary Table 1). During the sample collection, we carefully observed the field and selected only those plants that were healthy and fit among the infected plants. Furthermore, the isolated bacteria were tested against *U. virens,* and based on their biocontrol potential, the four most potent isolates have been selected and identified as bacillus species. There have been several studies that demonstrated the effective role of *Bacillus*-based bioformulations in mitigating or suppressing biotic stress in rice.

*U. virens* is a soil-borne phytopathogen that infects the roots of the rice plant and invades the plant system. The spores of the pathogen travel systemically through the stem of the plant and infect the panicle, leading to yield loss, seed sterility, and reduction in grain quality. Mature spores from the infected panicle fall on the soil during harvest, which serves as an inoculum to infect the rice plants in next season (Nguyen, 2012; Gupta et al., 2019). Experiment on biocontrol potential of four potent isolates showed good inhibition by all isolates, and among them, *B. vallismortis* showed the maximum inhibition zone with 44.6% inhibition. This is also confirmed by the SEM analysis of 10 days grown dual culture plate. Mycelial disintegration and broken mycelia were clearly visible in all four treatments (Sharma et al., 2019) (Figure 3). The possible reason behind this phenomenon could be the production of chitinase enzyme by these isolates. *Bacillus-*based bioformulation has chitinase production ability and produces various antagonistic secondary metabolites such as antimicrobial peptides, lipopeptides, bacteriocins, polyketides, and volatile organic compounds (VOCs) that directly inhibit the phytopathogen (Zhang et al., 2023). Our future study will focus on isolating these compounds to elucidate the effect of these metabolites. These metabolites are the key factors for biocontrol activity and the development of resistance against the pathogen. These VOCs, antimicrobial peptides, and other secondary metabolites participate in symbiosis and various signaling pathways related to plant immunity, disease suppression, growth and development.

After selecting the potent biocontrol agents against *U. virens*, increased attention was paid to identifying the major PGP traits, so that the role of isolates in growth promotion and plant fitness can be identified. Most of the PGPR isolates in this study possess most of the plant growth promotion traits that can be directly utilized by the plants, including phosphate solubilization, siderophore solubilization, zinc solubilization, ammonia production, and IAA (Hashem et al., 2019) (Tables 1, 2). Several studies have been conducted before on plant growth promotion by *Bacillus* isolates and antifungal activities, and based on this, many types of bioformulation are commercially available in the market (O’Brien, 2017; Rosier et al., 2018; Jamali et al., 2020; Kulkova et al., 2023). In general, these isolates match all the properties of biofertilizers. Previous studies show that *Bacillus* spp. actively solubilize the phosphate, solubilize inorganic phosphate to organic form, transfer to the host directly, and show a symbiotic relationship with plants (Bulgarelli et al., 2013). Three of the isolates, *B. subtilis*, *B. licheniformis* (BR_4, BU_7, and BU_8), were found as phosphate solubilizers. All isolates were found to be positive for IAA production, which is an essential signaling molecule that helps the interaction of microbes with the host (Olanrewaju et al., 2017; Matsuda et al., 2018). During the *invitro* PGPR test, all four isolates, namely, *B. subtilis*, *B. licheniformis* (BU_7 and BU_8), and *B. vallismortis* (KU_7), were depicted as, ammonium solubilizers (Table 1).

Metal ions, such as zinc, play a crucial role in redox reactions in biological systems. Zn is required as a co-factor for enzymes, such as SOD, that are involved in the suppression of ROS. Plants having Zn deficiency has also led to a reduction in photosynthesis and IAA, which ultimately leads to a reduction in crop yield (Singh et al., 2022a,b). Many *Bacillus* species are reported as zinc solubilizers (Masood et al., 2022). During this study, only one strain, *B. vallismortis* was found as a zinc solubilizer. Apart from Zn, these PGPR strains are also siderophore producers, which is an essential component for Fe sequestration in plants. Fe aids in the increase of chlorophyll content and chloroplast development in plants, which directly participate in crop yield by increasing the amount of photosynthate (Shahnaz and Manjurul, 2018).

Furthermore, in this study, *Bacillus*-based bioformulation is used to identify the role in growth promotion and to alleviate the biotic stress caused by *U. virens*. *Bacillus* colonizes the rhizosphere of the plant that secretes antagonistic metabolites, which protect the plant through direct and indirect mechanisms (Zhang et al., 2023). These PGPR-based bioformulations are prepared and applied in various ways such as seed treatment, foliar spray, root treatment, and soil drenches (Jaber and Ownley, 2018). These methods have shown promising results in reducing the disease incidence and severity in both *in vitro* and field conditions (Kantachote et al., 2016). In this study, we have used talc-based bioformulation to check the effect of biopriming on seed germination and under field conditions employing the root treatment method after testing it *in vitro*. Previous studies have also reported the use of *B. velengensis* Strain N1 for controlling leaf blight in rice (Sawatphanit et al., 2022). Comparable studies have employed the use of *T. harzianum*, *T. atroviride*, *B. subtilis,* and *B. amyloliquefaciens* in the biocontrol of false smut (Baite et al., 2022). Other than these strains, bacterial spp., such as *Bacillus, Streptomyces, and Pseudomonas* and fungal spp., such as *Trichoderma* and *Penicillium,* have been widely used as biofertilizers and biocontrol agents in agriculture (Kasotia et al., 2012; Lahlali et al., 2022).

**Figure 9:**
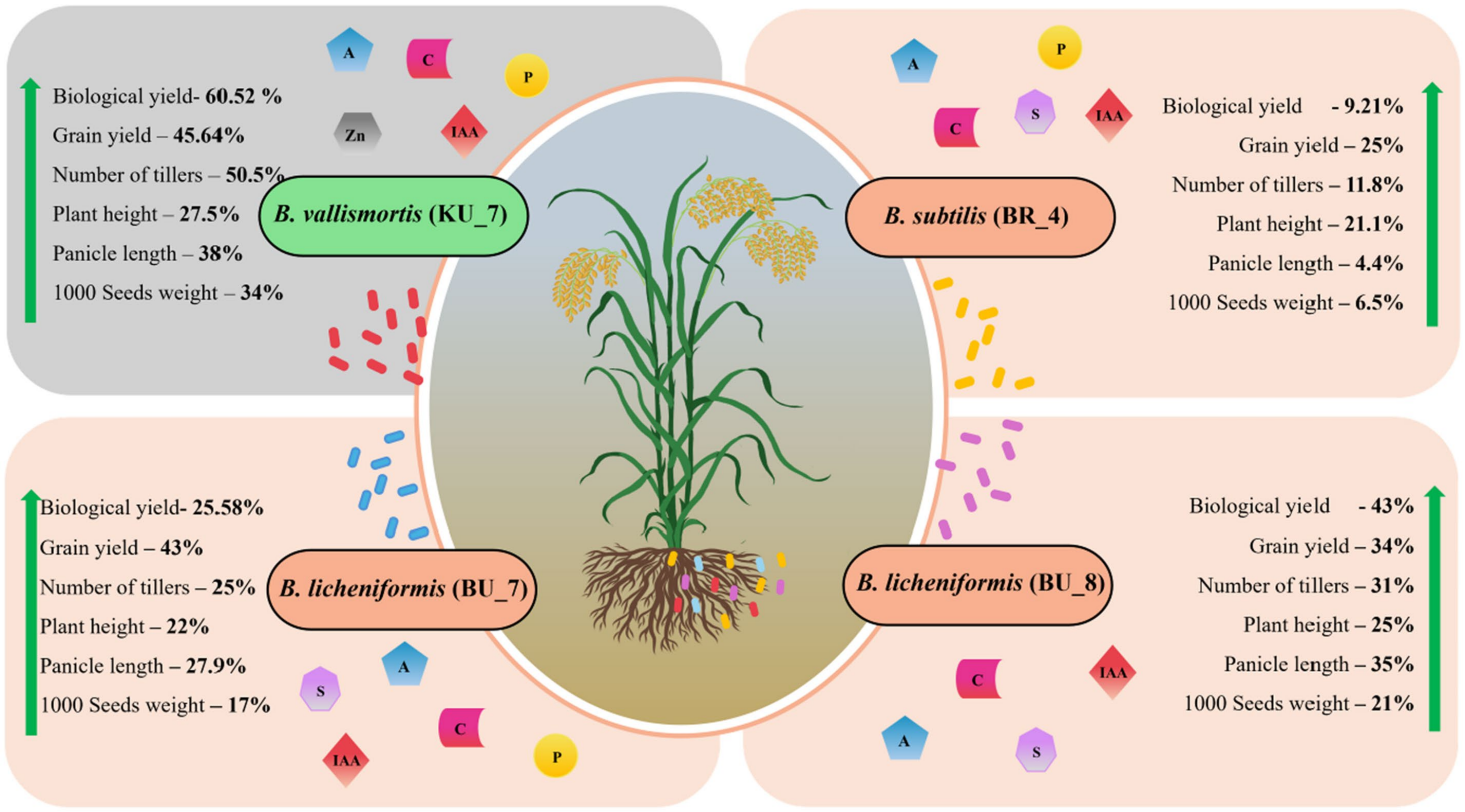
Diagrammatic representation of the overall increase in the agronomical parameters and crop yield using PGPR treatment with *B. subtilis* (BR_4), *B. licheniformis* (BU_7), *B. licheniformis* (BU_8), and *B. vallismortis* (KU_7). The figure depicts the average growth percentage of both years (2019 and 2020).

During the pot trials in green house conditions, we have observed the lower disease incidence and growth promotional effect by employing bacterial strains as a treatment. To observe the effect on large-scale and natural field conditions, field trials have been performed. Experimental trials were performed during the kharif crop season (June–October), and the field trials were performed consecutively for 2 years in 2019 and 2020. Root treatment with *B. licheniformis* (BU_7 and BU_8), *B. subtilis* (BR_4), and *B. vallismortis* (KU_7) strains individually resulted in enhanced agronomical parameters such as number of tillers, plant height, flag leaf length and width, panicle length, grain yield, and 1,000 seeds weight. Biological yield and total yield were also taken after the full harvest. Figure 9 represents the overall field parameters that are enhanced by the treatment of each bacterium. In our study, the highest biological and grain yields of 61 and 43% were observed, respectively, with the treatment with *B. vallismortis* (KU_7) (Figure 9). Similar results have been reported where treatment with other PGPRs promoted grains yield of 10–51% under greenhouse conditions and 4.8–9.2% increase in field conditions (Xiao et al., 2020).

Our isolates showed a higher production of total chlorophyll and total protein content in PGPR-treated plants, which may lead to enhanced crop production and biological yield. After the harvesting, the left paddy straw was used for fodder and other purposes, such as mushroom production and vermicomp*o*st (Figure 6). When plants are exposed to biotic stress, they exhibit oxidative damage by producing various types of reactive oxygen species (Padró et al., 2021). To protect from oxidative damage, the plant defense system produces some ROS scavenging enzymes, such as catalase (CAT) and superoxide dismutase (SOD). SOD and CAT are considered key enzymes for the first line of defense and act as biochemical markers in plants for oxidative damage along with the indication of the start of defense against any stress (Gill and Tuteja, 2010; Demidchik, 2015). These two enzymes critically mitigate the effect of oxidative damage in the cell. The result of this study showed an increased concentration of CAT and SOD as compared to the untreated control. In this study, stress-responsive parameters such as catalase and superOxide Dismutase activity, were checked and found to be enhanced in treated plants as compared to control (Figure 6).

To observe the efficiency of the isolated microbes in field conditions against *U. virens,* we have taken the highly susceptible cultivar HKR126 for the experiment. Disease incidence in this variety was reported up to 68.6% (Kumari and Kumar, 2015). This variety was also used by Sunder et al. (2005) in Haryana and resulted in 30–87% yield loss and a reduction in seed quality. Bag et al. (2021) stated that false smut infection in rice leads to an increase in the formation of false smut balls in the panicle and increases the count of unfilled grains or chaffiness. In this study, less disease incidence was observed in the HKR-126 rice variety, as reported by a previous study. According to the result, disease incidence ranged between 5–20% with all four bacterial treatments, while control showed 40% of infected panicles, while previous studies reported 5–85% of disease incidence in Tamil Nadu, India (Baite and Sharma, 2015; Sun et al., 2020).

Despite the potential benefits of *Bacillus*-based bioformulation for rice false smut disease management, the process can be influenced by several other factors, such as environmental conditions, soil composition, formulation composition, and genetic diversity of both plant and the pathogen, and it is crucial to optimize the conditions and application strategies in different agro-ecosystems to obtain the maximum efficacy of the product. Hence, they serve as a valuable tool in ensuring better rice yield and disease management (Pandey et al., 2024).

To enhance crop productivity and fulfil the global rice demand, farmers are continuously using chemical fertilizers without knowing the serious consequences in terms of soil and consumer health. Excessive use of chemical fertilizers may cause soil sterility and changes in the physiochemical properties. Continuous use of these fertilizers decrease the nutritional value of seeds, yield, and seed quality (Singh et al., 2014). Alternatively, rice false smut disease is controlled by the application of commercially available fungicides (trifloxystrobin with tebuconazole, propiconazole, and kresoxim methyl) at distinct stages of rice growth such as the booting stage, the panicle emergence stage, or the pre-flowering stage (Barnwal et al., 2016). Incessant application of fungicides causes resistance in the pathogen against fungicide over a period of duration and persistence in the environment. Fungicides, such as propiconazole, have led to the development of fungicide-resistant *U. virens* strains due to repeated exposure and cause environmental pollution (Zhou et al., 2019). This also contributes to increasing the cost of production and soil infertility.

PGPRs as bio-inoculants are the most alluring alternatives to chemical-based fertilizers as they have a positive impact on plant growth, soil, and the environment (Kantachote et al., 2016). The application of plant growth-promoting rhizobacteria (PGPR) that can serve as a biocontrol will not only enhance crop productivity but will also aid in the management of false smut disease. The present study is the few steps forward towards the microbial sustainable approach. Here, the isolates evaluated the efficacy of root treatment by the PGPR bioformulation, and these isolates possess antifungal activity against the rice false smut pathogen, *U. virens.* These microorganisms can be utilized as biofertilizers for better yield and at the same time protecting the plant from pathogens naturally.

Here, we have examined the effect of isolates on biocontrol and plant growth promotion properties individually. In the next phase, a consortium of potent microbes with different properties will be developed and its role in biocontrol activity along with growth promotion and secondary metabolites production will be assessed. The mechanism of false smut suppression will also be elucidated.

## 5 Conclusion

In modern times, chemical fertilizers and fungicides are being used for crop yield. These chemicals may increase crop productivity but are unable to increase seed quality and, at the same time show deleterious effects on human and soil health. These chemical fertilizers gradually mine the soil fertility and physiochemical properties, thus making the land barren and acidic. In this study, the bacteria identified from the rhizospheric soil were tested against *U. virens*, which causes rice smut disease. All four bacteria that belong to the *Bacillus* genus showed biocontrol activity and plant growth promoting traits, which led to higher rice yield in the pot and under field conditions. Among all isolates, *B. vallismortis* (Figure 9) showed the maximum inhibition of plant pathogens and several plant growth promotion activities. In field trials with *B. vallismortis* isolates, the incidence of rice smut disease was also lower than in other treatments. In this way, we conclude that the *B. vallismortis* species identified in this study help host plants to promote growth and enhance the seed quality naturally including all three isolates. This will also improve the quality of soil health and fitness naturally. The use of bioformulation can improve crop yield and overall productivity, leading to enhanced crop production and addressing the need for global food security. Meanwhile, bioformulations may reduce the need for synthetic fertilizers, which results in an impact on the emission of greenhouse gases and water pollution. Finally, we conclude that these PGPRs are a cost-effective and sustainable approach for rice cultivation.

## Data availability statement

The datasets presented in this study can be found in online repositories. The names of the repository/repositories and accession number(s) can be found in the article/Supplementary material.

## Author contributions

NP: Visualization, Conceptualization, Writing – original draft, Validation, Software, Methodology, Formal Analysis, Data curation. RV: Software, Formal analysis, Data curation, Writing – review & editing, Validation. AR: Methodology, Writing – review & editing. AS: Supervision, Conceptualization, Writing – review & editing, Visualization, Resources. SK: Project administration, Writing – review & editing, Visualization, Resources. RT: Validation, Formal analysis, Software, Writing – review & editing MK: Project administration, Resources, Writing – review & editing, Visualization, Validation, Supervision, Conceptualization. NS: Resources, Project administration, Funding acquisition, Validation, Supervision, Investigation, Conceptualization, Writing – review & editing, Visualization.
