# Supplementary material for: Exploring the potential of Bacillus for crop productivity and sustainable solution for combating rice false smut disease
Source: Front Microbiol. 2024 May 28;15:1405090. doi: 10.3389/fmicb.2024.1405090 (PMC11165134; doi:10.3389/fmicb.2024.1405090)
Supplement: Supplementary file 1 [file Data_Sheet_1.docx]

**Exploring the Potential of *Bacillus* for Crop Productivity and Sustainable Solution for Combating Rice False Smut Disease**

**Neha Pandey^1,2^, Richa Vaishnav^1^, Asha Singh Rajavat^1^, Arvind Nath Singh^3^, Sanjay Kumar^2^, Ravi Mani Tripathi^5^, Madan Kumar^4^, Neeraj Shrivastava^1*^**

**^1^**Amity Institute of Microbial Technology, Amity University, Noida 201313 Uttar Pradesh, India

**^2^**ICAR- Indian Institute of Seed Science, Maunath Bhanjan, 275103, Uttar Pradesh, India

**^3^**ICAR- Indian Institute of Vegetable Research, Varanasi, 221305, Uttar Pradesh, India

**^4^**ICAR- Indian Institute of Agricultural Biotechnology, Garhkhatanga, Ranchi, 834003, Jharkhand, India

**^5^**Amity Institute of Nanotechnology, Amity University, Noida 201313 Uttar Pradesh, India

Corresponding Author: *Dr. Neeraj Shrivastava ([neersar@gmail.com](mailto:neersar@gmail.com))

**Supplementary material**

**Supplementary Table 1.**

Samples were collected from the Different villages of Fatehpur district. Five sample sites were taken for soil sampling during the flowering stage when infection occurs. Rhizospheric soil samples were obtained from the affected rice field by selecting healthy, non-infected plants.

| Serial number | Fatehpur (25.9210° N, 80.7996° E)  Sampling sites (Village) |
| --- | --- |
| 1 | Takari (25.4785° N, 81.2285° E) |
| 2 | Dhata (25.5365° N, 81.2361° E) |
| 3 | Bhurchuni (25.5095° N, 81.2181° E) |
| 4 | Gopalpur (26.0419° N, 80.6466° E) |
| 5 | Bamhrauli (25.4616° N, 81.2161° E) |

**Supplementary figure**

**
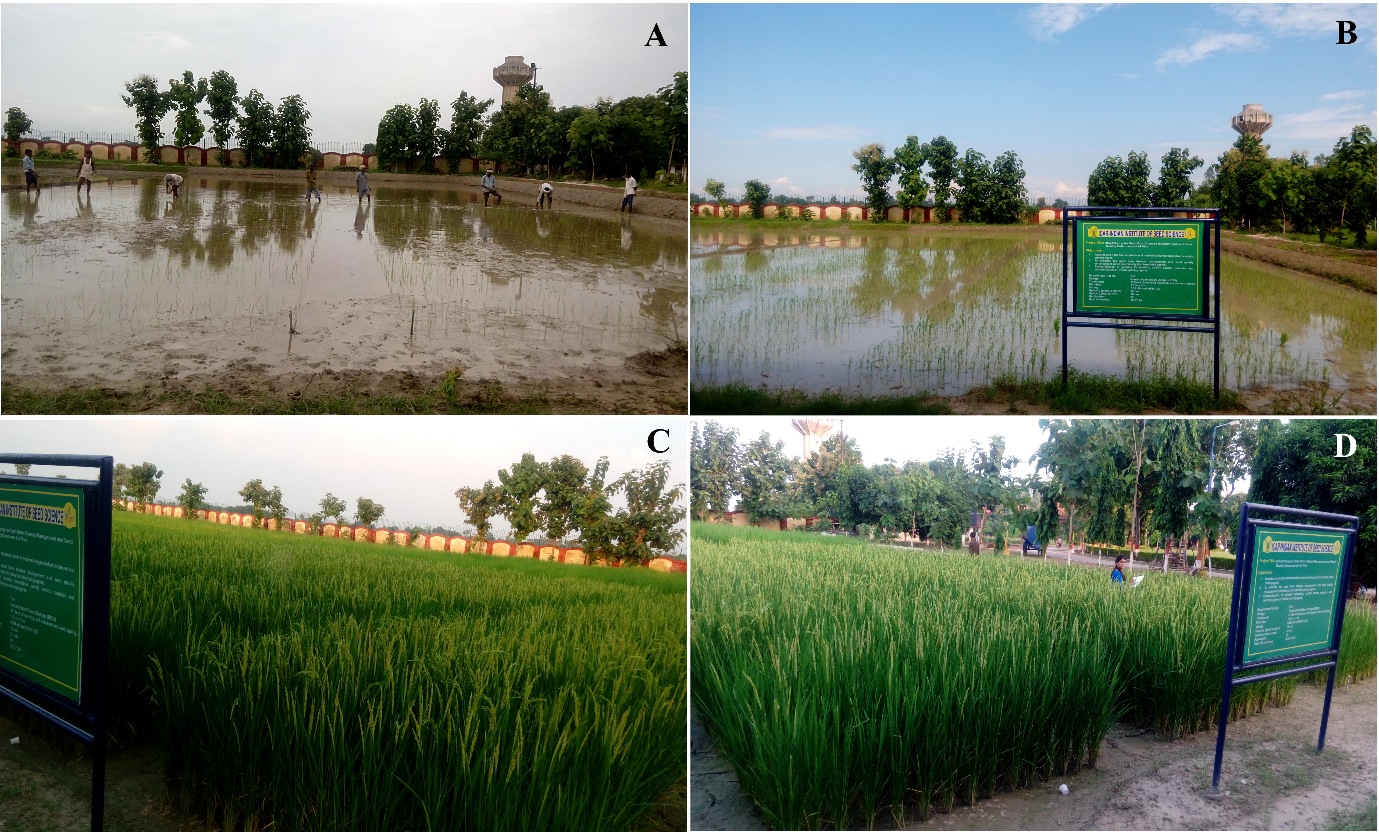
**

**Figure S1.** The field experiment set-up.

**(A)** Shows transplantation of root treated seedlings, **(B)** After the 10 days after the seedling settled in the field, **(C)** Picture taken during flowering, **(D)** Rows showing the replication numbers.
